# Social Disadvantage and Crime: A Criminological Puzzle

**DOI:** 10.1177/0002764216643134

**Published:** 2016-04-27

**Authors:** Per-Olof H. Wikström, Kyle Treiber

**Affiliations:** 1 University of Cambridge, Cambridge, UK

**Keywords:** crime, social disadvantage, social mechanisms, situational action theory

## Abstract

In this article, we analyze the relationship between social disadvantage and crime, starting from the paradox that most persistent offenders come from disadvantaged backgrounds, but most people from disadvantaged backgrounds do not become persistent offenders. We argue that despite the fact that social disadvantage has been a key criminological topic for some time, the mechanisms which link it to offending remain poorly specified. Drawing on situational action theory, we suggest social disadvantage is linked to crime because more people from disadvantaged versus affluent backgrounds develop a high crime propensity and are exposed to criminogenic contexts, and the reason for this is that processes of social and self-selection place the former more frequently in (developmental and action) contexts conducive to the development and expression of high crime propensities. This article will explore this hypothesis through a series of analyses using data from the Peterborough Adolescent and Young Adult Development Study (PADS+), a longitudinal study which uses a range of data collection methods to study the interaction between personal characteristics and social environments. It pays particular attention to the macro-to-micro processes behind the intersection of people with certain characteristics and environments with certain features – i.e., their exposure – which leads to their interaction.

‘Everybody believes that “poverty causes crime” it seems; in fact, I have heard many a senior sociologist express frustration as to why criminologists would waste time with theories outside the poverty paradigm. The reason we do… is that the facts demand it’.

Robert J. Sampson (2000: 711)

## Introduction

The role of social disadvantage (*the comparative lack of social and economic resources*) in crime causation is one of the most academically and publically discussed topics in crime causation. It is difficult to imagine any criminological topic that is more debated but less scientifically understood than the extent and nature of the relationship between social disadvantage and crime (e.g., Katz, 1988; Sampson, 2000, 2012; Tittle & Meier, 1990). While research findings generally suggest that social disadvantage (typically in reference to families and neighborhoods) is somehow implicated in crime causation, there is far from a simple one-to-one relationship, and researchers avidly disagree about the strength and nature of this relationship, with some even questioning whether there is a relationship at all (e.g., Agnew, 2001, 2006; Bjerk, 2007; Braithwaite, 1979, 1981; Brooks-Gunn, Duncan, & Aber, 1997; Coulton, Korbin, Su, & Chow, 1995; Dunaway, Cullen, Burton, & Evans, 2000; Duncan, Brooks-Gunn, & Klebanov, 1994; Elliott & Ageton, 1980; Fergusson, Swain-Campbell, & Horwood, 2004; Hay, Fortson, Hollist, Altheimer, & Schaible, 2007; Hindelang, Hirschi, & Weis, 1979; Hirschi, 1969; Janson & Wikström, 1995; Jarjoura, Triplett, & Brinker, 2002; Kornhauser, 1978; Loeber & Wikström, 1993; Messner & Rosenfeld, 1996; Reiss & Rhodes, 1961; Sampson, 1993, 2012; Shaw & McKay, 1969; Smith, 1991; Tittle, 1983; Tittle & Meier, 1990; Tittle, Villemez, & Smith, 1978; Wikström, 1990, 1991; Wikström & Butterworth, 2006; Wikström & Loeber, 2000; Wikström & Sampson, 2003; W. J. Wilson, 1987; J. Q. Wilson & Herrnstein, 1985; Wright et al., 1999).

Those who work with persistent offenders (and prisoners) on a regular basis are keenly aware that most come from disadvantaged backgrounds. In fact, any focus on offenders who hold (and warrant) the attention of the media, politicians, and practitioners (those who are more serious and/or frequent offenders) tends to validate the assumption that social disadvantage, as a common precursor, is a key cause of crime. This explains why many practitioners, policy makers, members of the general public, and even some academics perceive the relationship between social disadvantage and crime involvement to be strong and well-established. However, in focusing solely on offenders, an irreconcilable truth gets overlooked: *Although most persistent offenders come from disadvantaged backgrounds, most people from disadvantaged backgrounds do not become persistent offenders*. This fact may help explain why many people are convinced that social disadvantage is a main driver of crime, while research at best shows only a rather weak general (statistical) association between key indicators of social disadvantage and crime.

The main “criminological puzzle” (the key research question) is thus not why there is such a relatively weak (statistical) relationship between social disadvantage and crime (this is fairly well established) but why most persistent offenders come from a disadvantaged background, while most people from such backgrounds do not develop into persistent offenders. To answer this question requires a better understanding of the *mechanisms* through which social disadvantage is implicated in the development of persistent offending.

In this article, we propose to make some initial efforts to advance knowledge about the relationship between social disadvantage and crime involvement through the application of situational action theory (SAT) and the analysis of data from a random sample of U.K. adolescents from the longitudinal Peterborough Adolescent and Young Adult Development Study (PADS+).

SAT’s proposed explanation of the relationship between disadvantage and crime can be stated in three main hypotheses (see the theory section below):

**Hypothesis 1:** Differences between people in crime involvement are due to differences in their crime propensity and criminogenic exposure. SAT asserts that a person’s crime propensity is essentially a consequence of his or her personal morals and ability to exercise self-control, while a setting’s criminogeneity is a consequence of its moral context (its moral norms and their enforcement).
**Hypothesis 2:** Differences in crime involvement by disadvantage group are due to the fact that more people who grow up and live in disadvantaged circumstances develop a high crime propensity and are more frequently exposed to criminogenic settings.
**Hypothesis 3:** Differences in the number of crime prone people and the extent of their criminogenic exposure by disadvantage group are a consequence of disadvantage-related differences resulting from (rules and resource based) social and self-selection processes.

The first (situational) hypothesis has been thoroughly studied and supported in our previous research (see Wikström, Oberwittler, Treiber, & Hardie, 2012). In this article we will focus mainly on empirically exploring the second hypothesis that disadvantage-related differences in crime involvement are primarily due to disadvantage-related differences in the number of crime-prone people and their level of exposure to criminogenic settings. In other words, we assume that when controlling for crime propensity and criminogenic exposure, any predictive effect of disadvantage will vanish. If we are correct in this assumption, we assume that this is due to the fact that more people from disadvantaged backgrounds have been exposed to developmental contexts that promote the development of a stronger crime propensity and that more people in disadvantaged circumstances are exposed to criminogenic settings (Hypothesis 3). In this study, we can partially test the latter hypothesis by exploring if people from disadvantaged circumstances spend more time in criminogenic settings. However, we will not explore whether those with higher crime propensity have a history of exposure to developmental settings promoting a higher crime propensity. This assumption remains to be tested in future studies.

## Situational Action Theory

**Figure 1.**
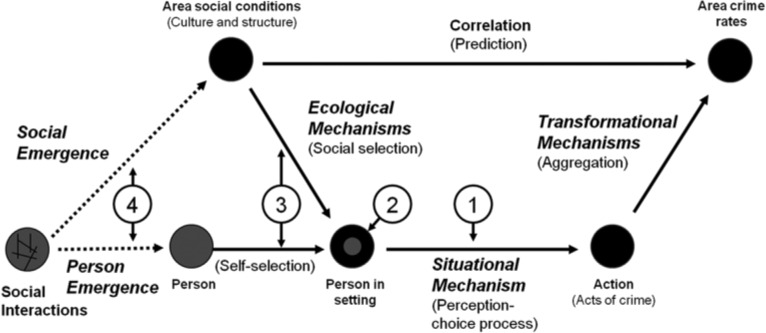
The theoretical framework of situational action theory. *Source:* Wikström, P-O. H. (2011). Does everything matter? Addressing the problem of causation and explanation in the study of crime. In J. McGloin, C. J. Sullivan, & L. W. Kennedy (Eds.), *When crime appears: The role of emergence* (pp. 53-73). London, England: Routledge.

SAT defines crime as acts that break rules of conduct stated in law and analyzes crime as moral actions; that is, as actions guided by rules about what actions are right or wrong under particular circumstances, with the law being seen as one among many sets of rules of conduct that guide people’s actions (e.g., Wikström, 2006, 2010). The framework of SAT is briefly summarized in the following key propositions and illustrated in Figure 1.

**Proposition 1:** Crime is ultimately an outcome of *a perception–choice process*.
**Proposition 2:** This perception–choice process is initiated and guided by relevant aspects of the person–environment interaction.
**Proposition 3:** Processes of social and self-selection place kinds of people (those with certain personal characteristics) in kinds of settings (those with certain environmental and circumstantial features), creating particular kinds of interactions.
**Proposition 4:** What kinds of people and what kinds of settings are present in a jurisdiction is the result of historical processes of personal and social emergence.

**Figure 2.**
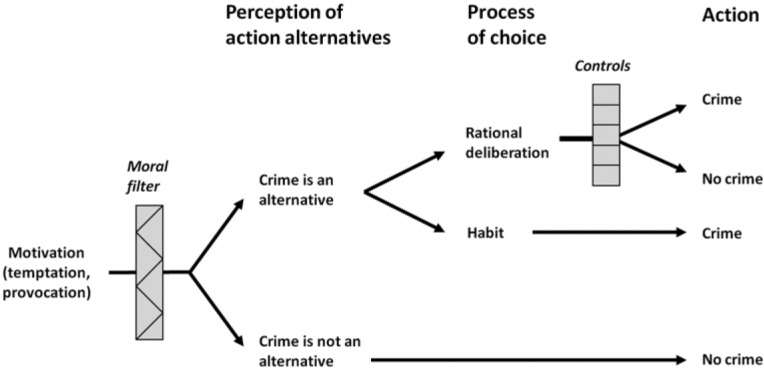
Key situational factors and processes in crime causation according to situational action theory. *Source*: Wikström, P-O. H. (2011). Does everything matter? Addressing the problem of causation and explanation in the study of crime. In J. McGloin, C. J. Sullivan, & L. W. Kennedy (Eds.), *When crime appears: The role of emergence* (pp. 53-73). London, England: Routledge.

Propositions 1 and 2 refer to the situational model, and Propositions 3 and 4 to the social model, of SAT. Figure 1 illustrates how these two models are linked. SAT proposes that the causes of action (such as acts of crime) are situational (Propositions 1 and 2) and that the social factors affecting people’s crime involvement (i.e., factors influencing processes of emergence and selection) are best analyzed as causes of the causes (Propositions 3 and 4). In essence, SAT argues that people commit crime because they come to see and choose (habitually or deliberately) an act of crime as an action alternative. The key situational factors and processes involved are illustrated in Figure 2. The basic idea is that *motivation* (temptations and provocations) initiates the action process (being a necessary but not sufficient factor), the *moral filter* (which depends on the interaction between personal morality and the moral norms of the setting) endorses action alternatives in response to a particular motivation, and *controls* (self-control and deterrence) influence the process of choice but only when the actor deliberates because there is conflicting rule guidance regarding crime as an action alternative (for details of the role of these factors and processes, see, e.g., Wikström, 2011; Wikström et al., 2012).

SAT argues that to understand how social factors (like social disadvantage) and developmental factors (such as cumulative experiences of disadvantage-related social conditions) influence people’s crime involvement as “causes of the causes” (i.e., causes of why people develop a high crime propensity and why settings develop weak law-relevant moral contexts), we need to understand how historical processes of *social and personal emergence* and contemporaneous processes of *social and self-selection* come to influence how people ultimately see their action alternatives and make their choices by exposing them to particular settings (environments) in which they develop and act (see also Wikström et al., 2012).

We posit that social disadvantage affects people’s crime involvement primarily through (rule- and resource-based) processes of social and self-selection which influence their exposure to crime-relevant developmental and action settings. We define *selection* as *social and personal forces (dependent on social and personal resources and rules) that enable (encourage or compel) or restrict (discourage or bar) particular kinds of people from taking part in particular kinds of developmentally and action-relevant time and place-based activities* (for more details, see Wikström et al., 2012). Specifically, we propose social and self-selection processes lead to young people from disadvantaged backgrounds being more profoundly exposed to (a) settings and circumstances which lead them to develop and sustain a high crime propensity (weak personal morality and a lack of ability to exercise self-control) and (b) moral contexts that are conducive to engagement in acts of crime (i.e., those in which rules of law are loosely applied and/or weakly enforced).

Thus, our proposed answer to the question, “What is the relationship between social disadvantage and crime?” is the following: *The impact of social disadvantage on young people’s crime may be primarily through disadvantage-induced selection processes which place disadvantaged young people more often than others in developmental contexts that are conducive to the development of a higher crime propensity, and in action contexts in which acts of crime tend to be encouraged (or at least are not strongly discouraged)*.

## The Peterborough Adolescent and Young Adult Development Study (PADS+)

**Figure 3.**
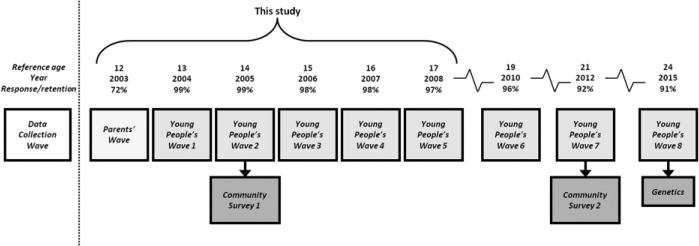
Overview of the PADS+ (Peterborough Adolescent and Young Adult Development Study) research design.

PADS+ is a longitudinal study that has followed a random sample of 716 young people who were living in the city of Peterborough in 2002, since they were 12 years old (2003), through adolescence, and now into young adulthood (Figure 3). Data used in this article were collected annually from the young people between 2004 and 2008, although additional waves have been completed at ages 19 (2010), 21 (2012), and 24 (2015). Methods included an extensive interviewer-led questionnaire, cognitive measures, a life events calendar, randomized scenarios, and a space–time budget. Data are also taken from an initial wave of data collected from participants’ parents in 2003 via a structured interview, including in-depth information about participants’ families’ social situations at the time of their enrolment in the study and retrospective information on their childhood experiences and critical life events. Over the period analyzed in this article, an exceptionally high retention rate was maintained, with 97% of the sample taking part in all five waves (ages 13-17). For this study, we focus on the 657 young people (92% of the sample) who took part in all five waves and completed space–time budgets in all five waves (for those who moved outside the study area, only time-budget data are available, which hinders some analyses). These young people were similar on all key variables, including crime involvement, except that those who were lost had significantly higher neighborhood disadvantage at age 12 (mean = 1.0 vs. 0.51, *p* = .001).

**Figure 4.**
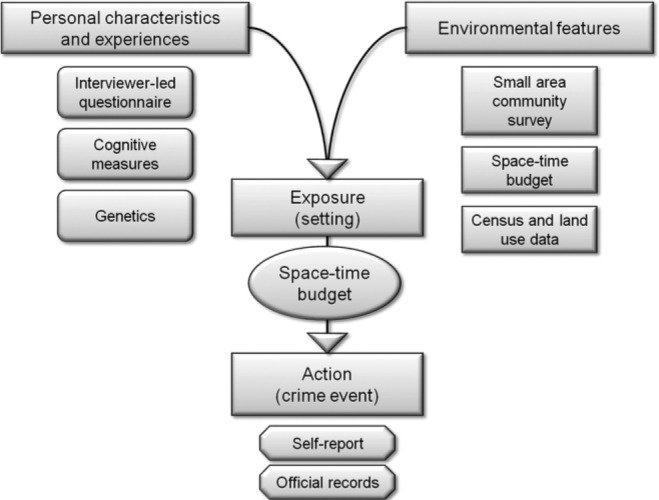
Overview of key PADS+ (Peterborough Adolescent and Young Adult Development Study) methodologies.

In addition to data collected from the main cohort study, two special small-area community surveys were carried out, one in 2005 and another in 2012, each with independent samples of around 6,000 randomly selected Peterborough residents aged 18 years or older, to gather data on social environments (e.g., levels of social cohesion and informal social control). This article draws on the 2005 survey, as well as external data from the 2001 U.K. Census. Data not analyzed in this article have also been collected from key social agencies (e.g., the police, probations service, schools, etc.). Figure 4 gives an overview of the PADS+ design and key methodologies; for a detailed presentation, including sampling methods and descriptives, data sources, and data quality, see Wikström et al. (2012).

In this article, we focus on the adolescent time window, which is of particular interest criminologically due to its encompassing most people’s entry into, escalation during and beginning of desistence out of, crime involvement. Adolescence is also particularly interesting in regard to changes in exposure to different social environments as young people shift their focus from family to peer activities and begin to establish their own autonomy through their social lives outside the home. Our hypothesis that social disadvantage influences young people’s crime involvement through its effects on the kinds of settings they are exposed to is highly relevant in the adolescent context, as it relates to differences in access to settings (e.g., what kind of settings are more proximate and young people’s mobility) as well as the kinds of settings they may choose to take part in (how they spend their time) as their autonomy increases. With five years of data on adolescents, including their experiences of social disadvantage, their changing activity fields and their patterns of crime involvement, PADS+ provides an excellent opportunity to study the selective influence of social disadvantage and its repercussions for young people’s social lives during adolescence, and on into young adulthood.

## Methods

### Creating a Baseline Measure of Childhood Social Disadvantage

Most studies of disadvantage focus on either family or neighborhood disadvantage. However, it has been noted that both capture important and distinct aspects of a person’s lack of economic and particularly social resources (e.g., Hay et al., 2007). We have accordingly developed a combined measure of family and neighborhood disadvantage using robust measures of each to comprehensively assess personal differences in early disadvantage.

#### Family Disadvantage

A *family disadvantage* index was constructed from three measures covered in the parents’ interviews, reflecting the family’s situation when the young people were 12 years old. It has been highlighted in previous research that young people are often not reliable informants of their family’s disadvantage (Duncan et al., 1994); by asking their parents, we acquire a much more accurate measure. We have included three key indicators in our family disadvantage measure: participants’ family’s household income,^1^ their parents’ highest educational level,^2^ and their parents’ highest occupational class^3^. Principal component analysis was employed to ensure these three variables represent one latent factor and factor scores were assigned to each participant.

#### Neighborhood Disadvantage

A *neighborhood disadvantage* index was constructed from 2001 U.K. Census data for the output area of the young person’s main home at the time of the parents’ interviews (2003), when participants were 12 years old. This measure is comparable with the Indices of Multiple Deprivation 2004 which measures disadvantage at a larger area level. This index included four items: the percentage of area residents who were working class,^4^ the percentage of area residents with no or low educational qualifications,^5^ the percentage of residents who were unemployed; and the percentage of area residents who resided in detached houses. Principal component analysis was employed to ensure these four variables represent one latent factor; as expected, all four loaded on a single factor and all loadings were greater than .50 (see Wikström et al., 2012, for details). Factor scores were assigned to each output area, and subsequently, each participant was assigned the factor score of their home output area.

#### Combined Disadvantage

**Figure 5.**
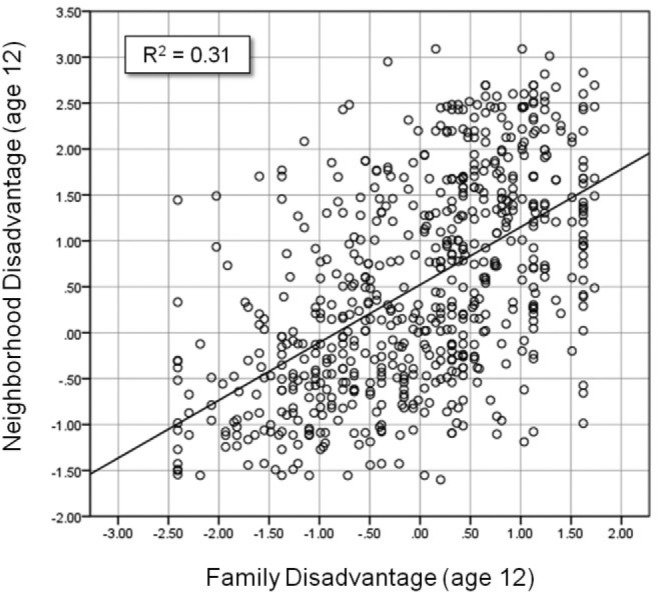
Scatter plot of family and neighborhood disadvantage.

Factor scores for family and neighborhood disadvantage were standardized (family scores across participants and neighborhood scores across output areas) and summed to create a *combined disadvantage* score for each participant, representing his or her experience of family and neighborhood disadvantage at age 12. For many analyses, combined disadvantage is divided into five equal-sized groups (20 percentiles). Family and neighborhood disadvantage were moderately correlated (Figure 5; *r* = .55). While the most disadvantaged families lived in areas with varying levels of neighborhood disadvantage, few of the most advantaged families lived in highly disadvantaged neighborhoods.

The combined family and neighborhood disadvantage scale has a reasonably approximately normal distribution and is consistent with other indicators of disadvantage measured in the parents’ interviews and an additional site survey conducted just after by PADS+ researchers. For example, families scoring highly on the combined disadvantage index were more likely at age 12 to live in a house whose interior, exterior, and surrounding area were in poor or very poor condition; to not be living in their family of origin; to have more biological and nonbiological siblings; to be an unplanned child; and to have a mother who suffered from postnatal depression.

### Measuring Social Environments and Activity Fields

One of the biggest shortcomings in criminology is a lack of adequate research into the role of social environments, driven in part by a lack of adequate data. There has been surprisingly little significant advancement in methods used to study environments in criminology since the works of the early Chicago School (e.g., Shaw & McKay, 1969; see Sampson, Morenoff, & Gannon-Rowley, 2002). The main exceptions are the recent introduction of large-scale community surveys (e.g., Sampson, Raudenbush, & Earls, 1997; Sampson & Wikström, 2008; Wikström, Torstensson, & Dolmen, 1997) and the development of ecometrics, a method for assessing the reliability of measures of environments, such as neighborhoods (Raudenbush & Sampson, 1999).

When social environments have been analyzed longitudinally, the focus has traditionally remained on the family and (to a lesser degree) school environments, ignoring neighborhoods (see, e.g., Brooks-Gunn, Duncan, Klebanov, & Sealand, 1993). The few recent longitudinal studies which have explored neighborhood effects (e.g., the Project on Human Development in Chicago Neighborhoods, or PHDCN) still overlook the role of people’s exposure to the wider environment. They equate a person’s environment only with his or her own neighborhood. However, findings from PADS+ show that people spend a lot of their time outside their neighborhoods and that people living in the same neighborhood may be exposed to very different kinds of environments (see, e.g., Wikström, Ceccato, Hardie, & Treiber, 2010). One reason studies fail to capture this variation in exposure is because they traditionally use a geographical unit of analysis which is too large and heterogeneous to adequately reflect the part of the environment that influences people’s actions and development (i.e., the part they experience with their senses); for example, most studies define neighborhoods using areas containing thousands of residents.

PADS+ was specifically designed to help overcome these limitations using new methodologies in combination with more established methods of measuring personal characteristics and experiences. These methods aim to

1. Measure the part of the environment which people directly experience (*using small-area units of analysis*)
2. Reliably measure relevant aspects of the social environment (*using ecometrics*; see Raudenbush & Sampson, 1999)
3. Account for the fact that people move around in space and encounter a wide range of different environments outside their neighborhoods (*using space–time budget methods*)

To measure young people’s exposure to different environments, PADS+ uses a strategy that combines (geographically matches) data from a small-area community survey (and official data on population composition and land use at the same small-area level) with data from a space–time budget (Wikström, Treiber, & Hardie, 2011). The community survey collects data from residents across the study area concerning social environmental variables such as social control and cohesion. These data are then linked geographically to data from the Census and other official databases to characterize areas using the smallest available unit, an output area (with, on average, 124 households), which, in turn, is linked to data from the space–time budget.

A *space–time budget* gathers very detailed time-diary data linked to a spatial unit and can therefore be used to calculate complex measures of exposure to (time spent in) a range of settings. The method includes hundreds of detailed codes for geographical locations, functional places (e.g., street corner), activities (e.g., skateboarding), and who a person is engaging with (e.g., peers) which combine to characterize a setting, as well as codes for additional circumstances including involvement in crime (as victim or offender) and substance use (see Wikström et al., 2012, pp. 70-75 and Technical Appendix A2, for details of these data). For each participant, PADS+ collects detailed space–time budget data about each hour over four days each wave (the Friday, Saturday, and two weekdays preceding the interview). Each wave comprises on average more than 65,000 hours of space–time budget data (amounting to more than 300,000 hours of data for the period analyzed in this article; 480 hours of data for each participant).

Data collected through these methodologies (space–time budgets combined with small-area community surveys) then allows the exploration of patterns of interaction between people’s exposure to different environments and their personal characteristics (e.g., using data from the interviewer-led questionnaires).

#### Criminogenic Exposure

Criminogenic exposure is a composite index of two scales: exposure to *criminogenic settings* and *peers’ crime involvement*.

Exposure to *criminogenic settings* is measured using space–time budget and social environmental data and refers to how many hours a person spent in *unstructured peer-oriented activities in local and city centers or areas with poor collective efficacy*.

*Peer-oriented activities* are defined as activities that take place outside of school and work settings in the presence of peers with no adult guardians present. Peers are frequently linked to crime involvement and may significantly influence crime involvement via their impact on the moral context (e.g., inducements to offend, relevant rules, and levels of enforcement). Of course peers can also strengthen the moral context, and this is taken into account by qualifying exposure to criminogenic settings according to levels of peers’ crime involvement (see below). Lack of *supervision* is a well-known predictor of crime involvement and can weaken the moral context particularly by reducing levels of enforcement.

*Unstructured* peer-oriented activities are peer-oriented activities which lack any goal-direction and mainly involve media consumption and socializing (see Wikström et al., 2012 for more details on this and other time use variables).

*Collective efficacy* refers to the capacity and willingness of people (typically residents) of a given area to act communally, for example, to hold and uphold a shared set of moral norms, and is supported by social cohesion and informal social control (Sampson et al., 1997; Sampson, Morenoff, & Earls, 1999). Areas with strong collective efficacy maintain and effectively enforce shared norms, while areas with poor collective efficacy are characterized by inconsistent norms and an inability to informally monitor and shape the conduct of area users. *Collective efficacy* is measured using two indices from the 2005 community survey in which residents living in each output area were asked about local social cohesion (five items tapping how much people in the neighborhood get along and share similar values) and informal social control (four items tapping the likelihood that neighbors would intervene if young people were breaking rules). Responses to these items were summed and averaged across respondents for each output area, then standardized and summated. See Wikström et al. (2012) for a description of reliability analyses used to validate this scale.

Commercial and entertainment *centers*, such as city and local centers, experience heavy traffic by nonresidents who move continuously in and out. The variety of setting users contributes to a lack of shared norms, and the sheer number of users reduces the ability for those users to monitor and control behavior; hence, the collective efficacy of area residents may not adequately reflect their moral contexts (see Wikström et al., 2012). Crime-relevant motivators, such as temptations which are presented by commercial venues and frictions which occur when different groups of people come into contact, especially in the presences of alcohol and other drugs, also tend to be concentrated in commercial and entertainment districts. Therefore, we separate these areas from residential areas in our analyses. *City and local centers* refer to a total of 10 output areas which comprise the main city center of Peterborough (four output areas) and four local centers (six output areas in total).

A composite exposure to *criminogenic settings* variable was created as the total number of hours recorded during the four days captured by the space–time budget in each wave which were spent in unstructured peer-oriented activities in local or city centers or in areas with poor collective efficacy.

*Peers’ crime involvement* was measured using a six-item scale asking if a person’s peers engaged in different acts of rule breaking (“no, never,” “yes, sometimes,” “yes, often [every month],” and “yes, very often [every week]”) including skipping school or work, getting drunk, substance use, shoplifting, vandalism, and fighting (see Wikström et al., 2012, for further detail). For each wave, participants received a summated peer crime involvement score.

The final measure of *criminogenic exposure* was created by standardizing participants’ criminogenic setting and peers’ crime involvement scores for each wave across all waves (3,000+ scores for each variable). Each participant’s five standardized scores for each variable were then summed to create a composite criminogenic exposure score for the entire period (ages 13-17). This scale was positively skewed, with most participants reporting less criminogenic exposure.

In some analyses, we refer to low, medium, and high criminogenic exposure. These categories are determined by standard deviations: high and low exposure refer to scores more than one standard deviation above and below the mean, respectively.

### Crime Propensity

Crime propensity represents an additive index of standardized scores on two scales: *personal moral rules* and *generalized ability to exercise self-control*.

*Personal moral rules* were measured by asking participants to rate whether they thought 16 acts of rule breaking were “very wrong,” “wrong,” “a little wrong,” or “not at all wrong.” These acts ranged from minor to more serious acts (e.g., teasing a classmate to breaking into a building to steal something). Details are provided in Wikström et al. (2012). Item responses were summed to create an index for each wave.

*Generalized ability to exercise self-control* was measured using eight items which asked participants to rate whether they “strongly agree,” “mostly agree,” “mostly disagree,” or “strongly disagree” with statements about themselves (e.g., “I never think about what will happen to me in the future”; “I lose my temper pretty easily”). This is a variant of Grasmick, Tittle, Bursik, and Arneklev’s (1993) scale which relies on items more consistent with SAT’s conceptualization of the expression of the ability to exercise self-control. As with the personal moral rules scale, self-control item responses were summed to create an index for each wave.

A composite crime propensity measure was then created by standardizing moral rules and self-control scores for each participant in each wave across all waves (3,000+ scores for each variable). Standardized scores for each variable from each wave were then summed to create crime propensity scores for each participant for the entire period (ages 13-17). Crime propensity was reasonably normally distributed across the study period.

As with criminogenic exposure, in some analyses, we refer to low, medium, and high crime propensity. These categories are determined by standard deviations: high and low propensity refer to scores more than one standard deviation above and below the mean, respectively.

### Crime

Crime involvement tapped into participants’ prevalence and frequency of engaging in nine acts of crime which are most representative of young people’s crime: arson, vandalism, theft from a person, shoplifting, assault, robbery, car crime (theft of or from a car), residential burglary, and nonresidential burglary. In all waves, participants were asked if they had committed the act during the previous calendar year and if so, how many times they had done so (for more details on these methods and general patterns of the sample’s crime involvement, see Wikström et al., 2012). From these items, prevalence and frequency measures were derived by wave and across all five waves (ages 12-16, as crime were reported for the previous year).

More than two thirds (70%) of the sample reported at least one act of crime during the study period, though most reported only one or two. Crime prevalences peaked at age 14, but for those who continued offending, crime frequencies increased steadily to age 16. A small proportion of the sample (4% or 27 young people) reported more than 100 acts of crime during the study period and was responsible for nearly half of all crimes reported (7,523 out of 15,970 crimes). These *persistent* offenders are specifically identified in some analyses. Assault was the most commonly reported act, with more than half of the sample reporting having hit or beaten up someone during the study period. For more details on the sample’s crime involvement between ages 12 and 16, see Wikström et al. (2012).

## Findings

*Are young people from disadvantaged backgrounds more heavily involved in crime?*

**Figure 6.**
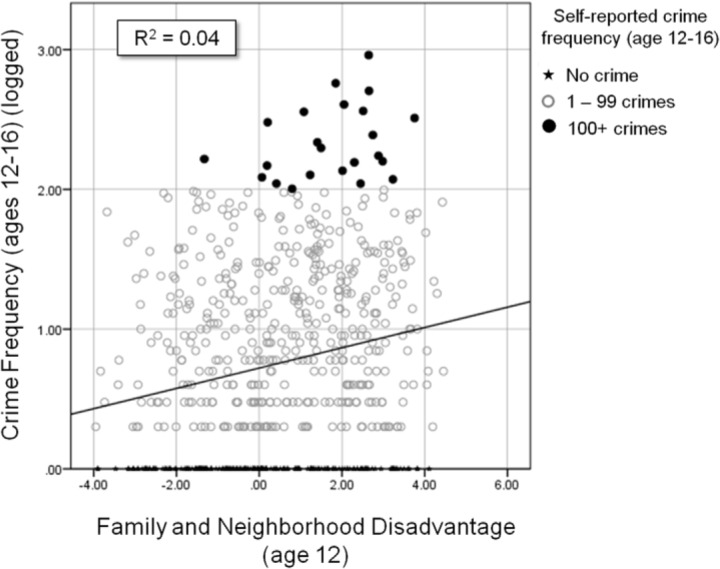
Scatter plot of combined family and neighborhood disadvantage at age 12 and crime frequency from ages 12 to 16 (logged).

The crucial answer is ‘not necessarily.’ As the distribution in Figure 6 shows, the vast majority of young people in the sample report few acts of crime, regardless of their preadolescent experiences of disadvantage, hence the correlation between disadvantage and crime involvement is decidedly small (*r* = .20 when crime is logged, .14 when not). Clearly, even a combined index of neighbourhood and family disadvantage is not good predictor of crime involvement, accounting for only 4% of the variance.

**Figure 7.**
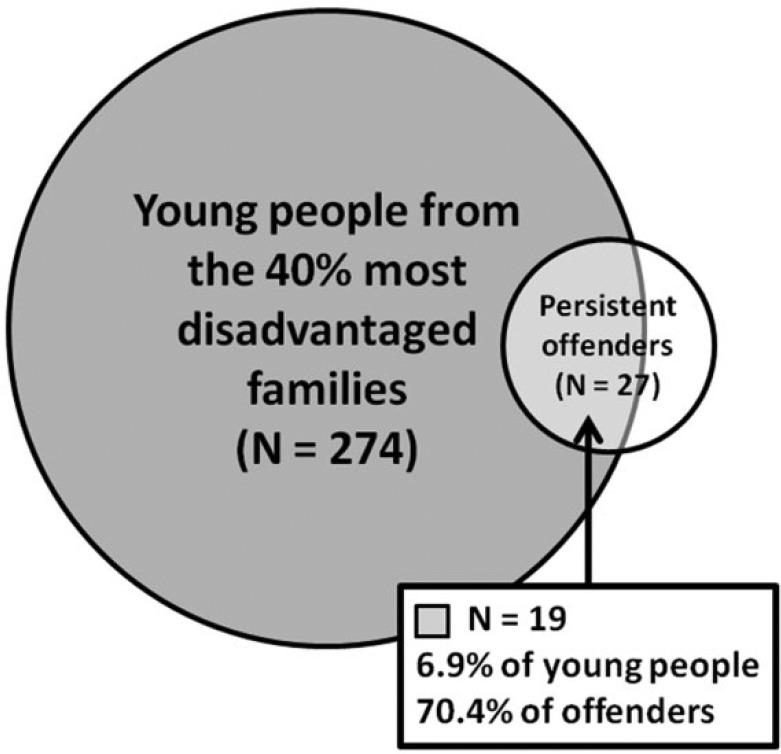
Venn diagram showing the overlap between young people from disadvantaged backgrounds and persistent offenders.

And yet PADS+ data also clearly illustrates the key criminological puzzle that was the impetus for this paper (Figure 7): a substantial majority (70.4%) of the sample’s persistent offenders came from disadvantaged backgrounds, but the majority of young people from disadvantaged backgrounds (93%) did not become persistent offenders.

*If disadvantage is not the answer, what is (Hypothesis 1)?*

Table 1 presents another illustration of the modest trend towards increasing crime prevalence and frequency with increasing social disadvantage by grouping participants into five equal sized (20 percentile) groups based on their disadvantage at age 12. If we compare these trends to those observed if the sample is divided instead into equal groups based on their crime propensity or criminogenic exposure, the key variables SAT proposes are directly linked to crime involvement (Hypothesis 1), we can see that the relationship between social disadvantage and crime pales by comparison.

*Do young people from disadvantaged backgrounds have higher crime propensity and more exposure to criminogenic settings (Hypothesis 2)?*

**Table 1.** Crime Involvement (Ages 12-16) by Participants’ Combined Disadvantage at Age 12, Crime Propensity, and Criminogenic Exposure (Divided Into Five Equal Groups).

	Percentage of offenders^a^	Mean crimes per offender^b^	Number of crimes	Percentage of crime	*N* (young people)	*N* (offenders)
*Combined disadvantage*						
Highest	77.9	46.5	4,748	34.0	131	102
High	77.1	36.5	3,682	26.3	131	101
Medium	73.2	27.5	2,638	18.9	131	96
Low	64.9	16.5	1,404	10.0	131	85
Lowest	56.5	20.4	1,510	10.8	131	74
All	69.9	30.5	13,982	100.0	655	458
*Crime propensity*						
Highest	94.6	73.3	9,021	64.4	130	123
High	90.0	25.2	2,951	21.1	130	117
Medium	77.1	13.2	1,331	9.5	131	101
Low	58.5	7.3	551	3.9	130	76
Lowest	30.8	4.0	158	1.1	130	40
All	70.2	30.7	14,012	100.0	651	457
*Criminogenic exposure*						
Highest	97.7	75.5	9,589	69.2	130	127
High	92.3	19.4	2,332	16.8	130	120
Medium	73.3	11.9	1,146	8.3	131	96
Low	55.7	8.1	591	4.3	131	73
Lowest	30.8	5.2	207	1.5	130	40
All	69.9	30.4	13,865	100.0	652	456

a For disadvantage groups, χ^2^ = 20.7, *p* < .000, and Cramer’s *V* = .18. Crime prevalence among the least disadvantaged is significantly lower than that of those with medium or higher disadvantage, while crime prevalence among those with high (or highest) disadvantage differs significantly only from those with low (or least) disadvantage. For propensity groups, χ^2^ = 169.6, *p* < .000, and Cramer’s *V* = .51, and for exposure groups, χ^2^ = 187.6, *p* < .000, and Cramer’s *V* = .54. All groups differ significantly in their crime prevalence except those with high and highest levels of crime propensity. ^b^Offenders’ crime frequency was significantly and linearly related to disadvantage, crime propensity, and criminogenic exposure group. However, the only significant differences by disadvantage group was between young people from low (and least) disadvantaged backgrounds and those from the most disadvantaged backgrounds, and only using a one-way Dunnett *t* test. All propensity groups differed significantly in their mean crime frequencies except the two groups who experienced the least disadvantage, and all exposure groups differed significantly except for those with low disadvantage and those with least or medium disadvantage.

**Figure 8.**
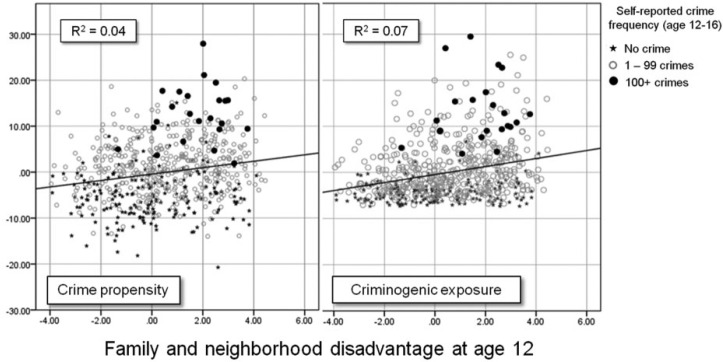
Scatter plot of participants’ combined family and neighborhood disadvantage at age 12 and crime propensity and criminogenic exposure between ages 13 and 17.

We have hypothesized that differences in crime involvement by disadvantage group are due to the fact that more people who grow up and live in disadvantaged circumstances develop a high crime propensity and are more frequently exposed to criminogenic settings. Table 2 shows this is indeed the case. On average, young people from disadvantaged backgrounds have a higher crime propensity (weaker personal morality and ability to exercise self-control; *r* = .19) and greater criminogenic exposure (more crime prone peers and exposure to criminogenic settings; r = .27) (see Figure 8).

**Table 2.** Mean Scores for Crime Propensity and Criminogenic Exposure (Ages 13-17) by Participants’ Combined Disadvantage at Age 12 (Five Equal Groups).

Combined disadvantage	Crime propensity		Criminogenic exposure	
	*M*	*N*	*M*	*N*
Highest	1.48	129	2.22	131
High	1.42	128	1.49	128
Medium	0.01	131	−0.11	131
Low	−1.31	131	−1.31	130
Lowest	−1.99	130	−2.34	130
All	−0.07	651	0.00	652
Correlation^a^	.19		.27	
*F* (sig)	.000^b^		.000^c^	
Linearity (sig)	.000		.000	

a Continuous variables. Both significant at the *p* = .000.^b^ The young people in lowest and low disadvantage groups had significantly lower crime propensities than young people in the high and highest disadvantage groups. ^c^Young people in the lowest disadvantage group had significantly less exposure than those in medium and higher disadvantage groups, young people in the low disadvantage group had significantly less exposure than those in the high and highest disadvantage groups, and young people in the medium disadvantage group had significantly less exposure than those in the highest disadvantage groups.

Although young people from disadvantaged backgrounds tend to have higher crime propensity and more criminogenic exposure on average, there is variation in both propensity and exposure at all levels of disadvantage, and the relationships between propensity and exposure and crime involvement remain strong regardless (Table 3 and Table 4). Importantly, there were no consistent substantive differences in crime involvement by disadvantage group among participants with different levels of crime propensity or criminogenic exposure (i.e., the effects of disadvantage on crime involvement appear to be mediated by propensity and exposure).

**Table 3.** Crime Propensity Groups (Ages 13-17) by Participants’ Combined Disadvantage at Age 12 (Five Equal Groups).

Combined disadvantage	Crime propensity												Correlation with crime frequency^a^	
	Low (>1 *SD* below the mean)				Medium (within 1 *SD* of the mean)				High (>1 *SD* above the mean)					
	%	*N*	*M*_prop_	*M*_crime_	%	*N*	*M*_prop_	*M*_crime_	%	*N*	*M*_prop_	*M*_crime_	*r*	ρ
Highest	8.5	11	−11.0	0.5	71.3	92	0.0	13.3	20.2	26	11.9	135.4	.75	.76
High	9.4	12	−10.5	1.2	68.0	87	−0.1	13.5	22.7	29	11.0	85.3	.68	.66
Medium	13.7	18	−9.3	0.7	71.8	94	−0.6	14.9	14.5	19	11.8	64.6	.61	.60
Low	22.1	29	−9.3	0.6	66.4	87	−0.4	8.4	11.5	15	9.0	43.6	.69	.68
Lowest	22.3	29	−10.4	0.6	68.5	89	−0.9	10.9	9.2	12	9.9	43.3	.68	.66
All	15.2	99	−9.9	0.7	68.5	450	−0.4	12.2	15.5	102	11.0	82.7	.70	.69
*F* (sig)			*ns*	*ns*			*ns*	*ns*			.007^b^	*ns*		
Linearity (sig)			*ns*	*ns*			*ns*	*ns*			.01	*ns*		

a Pearson’s correlation with logged crime frequency; Spearman’s rho with raw crime frequency. ^b^The mean crime propensity of those with low levels of disadvantage was significantly lower than the mean crime propensity of those with medium and highest levels of disadvantage (*p* < .05).

**Table 4.** Criminogenic Exposure Groups (Ages 13-17) by Participants’ Combined Disadvantage at Age 12 (Five Equal Groups).

Combined disadvantage	Criminogenic exposure													
	Low (>1 *SD* below the mean)				Medium (within 1 *SD* of the mean)				High (>1 *SD* above the mean)				Correlation with crime frequency^a^	
	%	*N*	*M*_exp_	*M*_crime_	%	*N*	*M*_exp_	*M*_crime_	%	*N*	*M*_exp_	*M*_crime_	*r*	ρ
Highest	7.6	10	−6.4	0.1	74.8	98	0.2	14.8	17.6	23	14.7	143.2	.61	.59
High	9.4	12	−6.2	1.1	75.0	96	0.1	13.8	15.6	20	13.0	110.4	.56	.54
Medium	10.7	14	−6.1	2.3	76.3	100	−1.4	13.0	13.0	17	12.7	76.9	.56	.54
Low	4.6	19	−6.2	0.5	78.5	102	−1.4	10.1	6.9	9	10.2	38.6	.46	.47
Lowest	20.8	27	−6.4	0.7	76.9	100	−1.8	12.2	2.3	3	14.6	90.3	.46	.47
All	12.6	82	−6.3	0.9	75.8	498	−0.9	12.8	11.0	72	13.2	103.1	.56	.54
*F* (sig)			*ns*	*ns*			.000^b^	*ns*			*ns*	.003^c^		
Linearity (sig)			*ns*	*ns*			.000	*ns*			*ns*	*ns*		

a Pearson’s correlation with logged crime frequency; Spearman’s rho with raw crime frequency. ^b^Young people who came from high and highest disadvantaged backgrounds had significantly more exposure to criminogenic settings than young people from less disadvantaged backgrounds. ^c^The only significant difference was between those with least and low disadvantage and was probably driven by the very low *N*.

*Is social disadvantage a cause of the causes of crime?*

The suggestion that the relationship between social disadvantage and crime involvement is fully mediated by differences in young people’s crime propensity and exposure to criminogenic settings (Hypothesis 2) is borne out by the regression models shown in Table 5. Disadvantage at age 12 significantly predicts crime involvement between ages 12 and 16, but explains very little of the variance in crime frequency (3.9%); by contrast, propensity and exposure together explain 55.6% of the variance, and fully mediate the effects of both family and neighbourhood disadvantage.

*How do activity fields differ for young people from disadvantaged backgrounds (Hypothesis 3ish)*?

**Table 5.** Disadvantage, Propensity, and Exposure Predicting Crime Involvement (Number of Crimes Logged).

	Model 1		Model 2		Model 3	
	β	*p*	β	*p*	β	*p*
Combined disadvantage	.20	.000	—	—	.02	*ns*
Propensity	—	—	.44	.000	.44	.000
Exposure	—	—	.37	.000	.37	.000
*R*^2^ × 100	—	3.9	—	55.6	—	55.5

Family disadvantage	.08	*ns*	—	—	.01	*ns*
Neighborhood disadvantage	.15	.001	—	—	.02	*ns*
Propensity	—	—	.44	.000	.44	.000
Exposure	—	—	.37	.000	.37	.000
*R*^2^ × 100	—	3.8	—	55.6	—	55.5

We have suggested that differences in the number of crime prone people and the extent of their criminogenic exposure by disadvantage group is a consequence of disadvantage-related differences in social and self-selection which expose more young people from disadvantaged backgrounds to more criminogenic developmental and action contexts (Hypothesis 3). Although we will not test these processes directly, we can preliminarily assess differences in exposure to settings and circumstances and shed light on these processes in action (Table 6).

**Table 6.** Participants’ Time Use (Ages 13-17) by Their Combined Disadvantage at Age 12 (Five Equal Groups).

Combined disadvantage	Hours spent (out of 480)					
	Unsupervised	Peer-oriented	Unstructured	In areas with poor collective efficacy	In city and local centers	Unstructured peer-oriented activities in city and local centers and areas with poor collective efficacy
Highest	71.0	41.4	132.5	133.6	23.3	11.6
High	69.6	38.2	127.7	90.9	30.8	9.1
Medium	68.5	36.8	116.8	57.0	25.3	6.1
Low	59.9	29.5	115.2	26.1	28.3	3.8
Lowest	58.7	27.7	104.3	21.9	24.9	3.3
All	65.6	34.8	119.3	66.1	26.5	6.8
*r* ^a^	.16	.21	.35	.54	*ns*	.29
ρ	.17	.20	.34	.55	*ns*	.30
*F* (sig)	.000^b^	.000^c^	.000^d^	.000^e^	*ns*	.000^f^
Linearity (sig)	.000	.000	.000	.000	*ns*	.000

a Using continuous variables. All correlations significant at the *p* = .000 level. ^b^Young people from 40% least disadvantaged backgrounds spent significantly less time unsupervised that those from 40% most disadvantaged backgrounds, with those from the 20% least disadvantaged backgrounds also spending less time unsupervised than those from the medium quintile. ^c^Young people from the 40% least disadvantaged backgrounds spent significantly less time in peer-oriented activities than young people from any other quintiles. ^d^Time spent in unstructured activities differed significantly between all quintiles except low and medium and high and highest. ^e^Time spent in areas with poor collective efficacy differed significantly between all quintiles except the low and lowest disadvantage. ^f^Young people from the 40% least disadvantaged backgrounds experienced significantly less exposure to criminogenic settings than young people from the 40% most disadvantaged backgrounds. Those from middle quintile of disadvantage also had significantly lower exposure than those from the 20% most disadvantaged backgrounds.

One very significant difference in time use by disadvantage groups relates to the time they spend in educational activities; those from disadvantaged backgrounds spend significantly less time in educational activities and at school than those from more advantaged backgrounds (for example, those in the highest disadvantage group spent, on average, nearly 1.5 hours less per day in educational activities – e.g., attending classes, doing homework – across the study period than those in the lowest disadvantage group). Substantive differences are evident at every age, though the most dramatic differences appear once the young people have reached the age at which they may leave compulsory education.

By contrast, young people from disadvantaged backgrounds spend more time on average in leisure activities, including socialising (close to one hour more per day across the study period), than young people from more advantaged backgrounds, and more of this time is unstructured. Disadvantaged young people also spend more time on average unsupervised, and in particular unsupervised with their peers (nearly half an hour more per day), and those peers are more likely to be crime prone.

Young people from disadvantaged backgrounds also spend considerably more time on average in areas with poor collective efficacy and less in area with strong collective efficacy. This is in part driven by the area characteristics of their home neighbourhoods, but disadvantaged young people also spend twice as much time outside their home neighbourhoods in areas with poor collective efficacy (two hours on average per day). They do not, however, spend much more time in the city and local centres. Overall, socially disadvantaged young people’s exposure to settings theorized to be conducive to crime is significantly higher on average than that of those who are less disadvantaged. However, it is important to note that this still represents only a fraction (less than 5%) of their time awake.

*Do young people from disadvantaged backgrounds who do not offend have lower crime propensity and less criminogenic exposure than those who do*?

We have drawn attention to the fact that not all young people from disadvantaged backgrounds commit acts of crime. If the explanation we have posited is correct—that is, disadvantage influences crime involvement via selection processes which lead to higher crime propensity and greater exposure to criminogenic settings—we would expect that these effects are attenuated for young people who come from disadvantaged backgrounds who do not offend. This is precisely what we find (Table 7). Young people from disadvantaged backgrounds who do not offend demonstrate average levels of personal morality and ability to exercise self-control more consistent with young people from the least disadvantaged backgrounds. Their time use as well is dramatically different from that of those from disadvantaged backgrounds who are crime involved, as is their peers’ crime involvement; they spend less time unsupervised with their peers and in areas with poor collective efficacy, and their peers are much less frequently involved in crime. These effects are not explained by differences in their experiences of disadvantage; they do not differ in their average family or neighborhood disadvantage or experience significant changes in their situations during the study period.

**Table 7.** Comparison of means for offenders and non-offenders with similar experiences of disadvantage.

	20% most disadvantaged		20% least disadvantaged	
	Non-offenders	Offenders	Non-offenders	Offenders
Crime propensity	−5.9	3.4	−5.8	0.9
Criminogenic exposure	−3.2	3.8	−4.5	-0.6
**Time use**				
Unsupervised	52.3	76.4	48.9	66.1
Peer-oriented	26.7	45.5	20.8	33.0
Unstructured	114.9	137.5	97.5	109.5
Poor collective efficacy	107.3	141.0	20.8	22.8
City or local centres	22.7	23.5	16.9	31.0
Unstructured peer-oriented activities in centres or areas with poor collective efficacy	4.3	13.7	1.6	4.6

A complementary assumption which can be drawn is that young people in the least disadvantaged areas who do offend would likewise have a higher crime propensity and criminogenic exposure than their nonoffending peers. We find this is also the case, although the differences are perhaps not as extreme as those between offending and nonoffending young people from disadvantaged backgrounds. Interestingly, one difference which arises is that while disadvantaged young offenders and nonoffenders differed in their exposure to areas with poor collective efficacy, but not significantly to city and local centers, the opposite is true for advantaged young offenders and nonoffenders; offenders from the least disadvantaged backgrounds spent significantly more time in the city and local centers than their nonoffending peers, but no more time in areas with poor collective efficacy. These differences in time use have interesting implications for understanding the impact of differences in mobility and access to settings between advantaged and disadvantaged young people, as well as for differences in the kinds of settings and circumstances which they may find crime conducive.

*Are young people from disadvantaged backgrounds more likely to have a high propensity AND high criminogenic exposure? Do young people from all backgrounds with a higher crime propensity AND criminogenic exposure commit more crime?*

**Figure 9.**
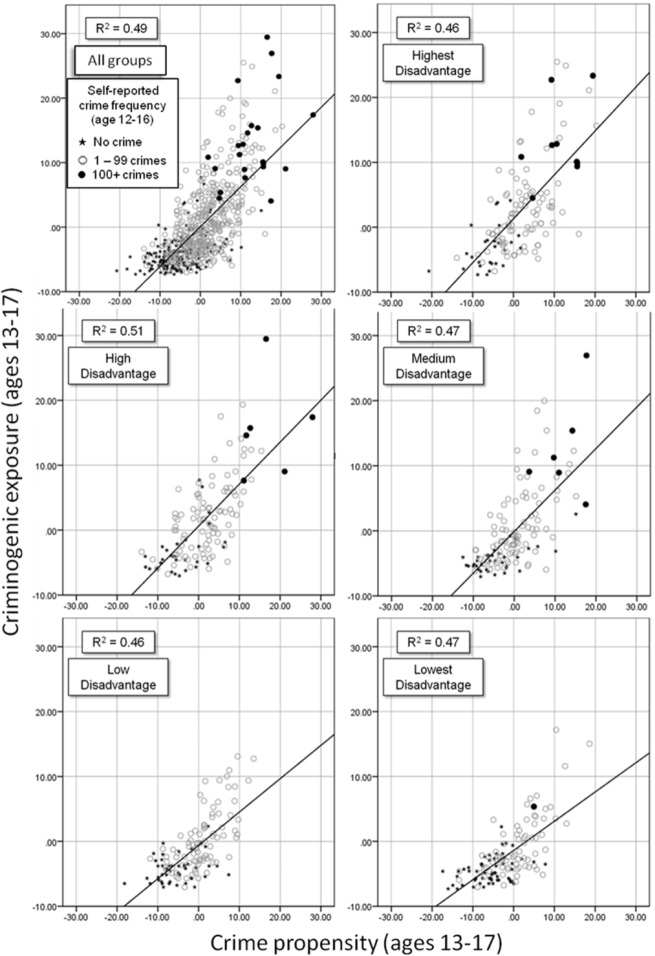
Scatter plots of crime propensity and criminogenic exposure (ages 13-17) by combined disadvantage (total and for five equal groups).

The main premise of SAT is that it is the interaction between crime propensity and criminogenic exposure that is the key to understanding crime involvement. The final analysis focuses in on this interaction. Table 8 shows that, as expected, more young people from disadvantaged backgrounds demonstrate a high crime propensity and experience a high rate of exposure to criminogenic settings than those from less disadvantaged backgrounds. However, regardless of their levels of disadvantage, young people with a high crime propensity and high criminogenic exposure report high rates of crime involvement (practically 100%) and extremely high crime frequencies. Figure 9 illustrates this pattern very clearly. The relationship between crime propensity and criminogenic exposure and crime involvement are remarkably similar across disadvantage groups: regardless of levels of disadvantage, as propensity and exposure increase, crime involvement increases (and the amount of explained variance is practically identical). The only substantial difference is that at lower levels of disadvantage, fewer young people experience a high crime propensity and high criminogenic exposure.

**Table 8.** Cross-Table of Crime Propensity and Criminogenic Exposure (Ages 13-17; Groups by *SD*) Split by Combined Disadvantage (Five Equal Groups) Showing Distributions and Crime Involvement.

Crime propensity	Combined disadvantage	Exposure											
		Low (>1 *SD* below the mean)				Medium (within 1 *SD* of the mean)				High (>1 *SD* above the mean)			
		*N*	%	Percentage of offenders	Mean crime frequency	*N*	%	Percentage of offenders	Mean crime frequency	*N*	%	Percentage of offenders	Mean crime frequency
Low (>1 *SD* below the mean)	Lowest	12	9.3	16.7	0.3	17	13.2	29.4	0.8	0	0.0		
	Low	10	7.7	0.0	0.0	19	14.6	31.6	0.9	0	0.0		
	Medium	4	3.1	25.0	0.3	14	10.7	35.7	0.8	0	0.0		
	High	5	4.0	40.0	1.2	7	5.6	28.6	1.1	0	0.0		
	Highest	4	3.1	0.0	0.0	7	5.4	42.9	0.9	0	0.0		
Medium (within 1 *SD* of the mean)	Lowest	14	10.9	14.3	1.1	74	57.4	71.6	12.9	0	0.0		
	Low	9	6.9	44.4	1.1	75	57.7	77.3	8.9	2	1.5	100.0	18.5
	Medium	9	6.9	33.3	1.7	78	59.5	80.8	10.0	7	5.3	100.0	85.9
	High	7	5.6	28.6	1.0	74	59.2	79.7	13.2	4	3.2	75.0	15.8
	Highest	6	4.7	16.7	0.2	78	60.5	82.1	11.9	8	6.2	100.0	36.8
High (>1 *SD* above the mean)	Lowest	0	0.0			9	7.0	88.9	27.7	3	2.3	100.0	90.3
	Low	0	0.0			8	6.2	87.5	43.0	7	5.4	100.0	44.3
	Medium	1	0.8	100.0	16.0	8	6.1	75.0	63.0	10	7.6	100.0	70.7
	High	0	0.0			12	9.6	100.0	26.3	16	12.8	100.0	134.0
	Highest	0	0.0			11	8.5	100.0	47.4	15	11.6	100.0	200.0

## Conclusions

The relationship between social disadvantage (the comparative lack of social and economic resources) and crime has been a persistent puzzle for criminologists. Consistent with most previous research, we have found that coming from a disadvantaged background was not a strong predictor of crime involvement in our sample, even when using a combined measure of family and neighbourhood disadvantage, and yet a large proportion of our most persistent offenders did indeed come from disadvantaged backgrounds.

We have presented, and tested, a new explanation for this seeming contradiction. First we have suggested that differences between people in their crime involvement are due to differences in their crime propensity and criminogenic exposure, and we have shown that crime propensity and criminogenic exposure are stronger and more consistent predictors of crime involvement than social disadvantage which fully mediate the relationship between social disadvantage and young people’s crime.

Second, we have suggested that differences in crime involvement by disadvantage group are due to the fact that more people who grow up and live in disadvantaged circumstances develop a high crime propensity and are more frequently exposed to criminogenic settings. We have shown this to be the case. We have also shown that the relationship between crime propensity, criminogenic exposure and crime involvement remains remarkably consistent and robust regardless of participants’ levels of disadvantage.

Finally, we have suggested that these differences in the number of very crime prone people and the extent of their criminogenic exposure by disadvantage group are a consequence of disadvantage-related differences resulting from (rules and resource based) social and self-selection processes. We have shown that young people’s time use differs significantly in criminogenic ways depending on their levels of disadvantage supporting our selection hypothesis. In future publications we will further explore the selection processes which influence young people’s activity fields, both in relation to developmental and action contexts, with the aim of better understanding why people vary in their crime propensity and criminogenic exposure, including what environmental qualities other than social and economic resources may explain such variations.

Our overall conclusion is that social disadvantage is only moderately related to factors related to crime involvement (crime propensity and criminogenic exposure). Our findings support the assertion that the relationship between social disadvantage and crime involvement may be explained by the fact that more young people who experience childhood disadvantage at home and in their neighbourhoods are likely to develop a high crime propensity and be exposed to criminogenic settings, but that these are far from inevitable outcomes of growing up disadvantaged.

## Notes

1. Before taxes; increments of £5,000 up to £70,000+.

2. University degree (three levels: doctorate, graduate, or undergraduate); A levels (or vocational qualifications such as a BTEC [Business and Technical Education Council] extended diploma or HND [Higher National Diploma]); O levels (compulsory education); not completed compulsory education.

3. A four-class scale divided into lower working (unskilled workers), working (skilled workers and low-ranking white-collar employees), lower middle (officials and small-scale entrepreneurs), and upper middle and upper (large-scale entrepreneurs, high-ranking officials, and high-ranking white-collar employees).

4. National Readership Survey.

5. National Qualifications Framework.
